# Note on Salicylic Acid

**Published:** 1875-04

**Authors:** Edward R. Squibb

**Affiliations:** Brooklyn, N. Y.


### MISCELLANEOUS.

### Note on Salicylic Acid.

BY EDWARD R. SQUIBB, M. D. OF BROOKLYN, N. Y.

This substance, long known as a rare and curious chemical derived from the vegetable kingdom, has lately been brought into prominent notice, chiefly in Germany, from its relations to those changes which are commonly known and best understood as fermentations, to which class or kind of changes so many diseases and pathological conditions are now pretty well known to belong.

The writer knows far too little of the subject and its relations to attempt an accurate or exhaustive paper upon it, and the object of this note is simply to call attention to it, that it may be read up in the current literature—to give a brief outline of its bibliography, that reference may be made in regard to its history—and to offer some thoughts in regard to its sphere in medicine.

Salicin is a glucoside or neutral vegetable principle discovered by Leroux* in 1830, in the bark of some species of willow, Salix, whence its name. It was afterward found in various species of poplar†, and in other trees and plants. Salicin was chiefly investigated by Piria‡ who gave an elaborate account of its derivatives, and among these, of salicylic acid. Early in its history the acid was prepared by Lœwig and Weidmann§ from the flowers of Spiræe ulmaria; and later, a research by Prof. Procter‖ of Philadelphia, showed that our oil of wintergreen, Gaultheria procumbens, was really a salicylous ether; and from this source salicylic acid was obtained by Cahours**. Gerhardt††, Ettling‡‡ and others contributed to the researches by which the properties and reactions of salicylic acid were accurately determined and its composition fixed; but as yet it was but a chemical curiosity whose potential possibilities were quite unknown. It still belonged to that class of substances which had simply consumed a large amount of patient labor, and in relation to which the rigid utilitarian asked Michael Farrady "What is the use of such things?" and received for reply the answer, "What is the use of a baby?"

The physiological and pathological effects of salicin, though imperfectly investigated, seems to have gradually and slowly directed attention to those of its derivatives, and occasional paragraphs have appeared in current scientific literature, from time to time, upon salicylic acid for some years past. But only within a year or two —and the writer regrets that he does not know by whom first—German writers have alluded to its peculiar and powerful effects as an antiferment and antiseptic. As its peculiar powers were recognized, and its importance became possible and probable, the sources from which it had been obtained as a chemical curiosity became impracticable, in consequence of the small quantity which could be obtained from them, and the great cost in material and labor.

The next step in the progress of salicylic acid toward practical utility affords an excellent illustration of the progress in chemical knowledge made of late years.

The modern chemist seems to know, within certain limits, the combinations of the elements in organic substances very much as he knows the axes of crystals, and hence deduces their planes of cleavage. That is, he knows how they will split up under given

---

* Journ. de Chim. Med. T. 6, F. 341. † Braconnot, Ann. Chim. Phys. T. 44, F. 296. ‡ Compt. Rend. T. 6, F. 388; Ann. Pharm. T. 30, F. 165.

§ Jour. pr. Chem; Bd. 19, S. 236. ‖ Amer. Jour. Pharm., v. 14, p. 211. ** Compt. Rend. T. 16, F. 863. †† N. Ann. Chim. Phys., T. 7, F. 217. ‡‡ Ann. Pharm. T. 53, F. 77.

conditions, and what new arrangements of their elements are possible or even practicable. And farther, he knows, by pure reasoning upon facts, what new elements to introduce between the molecules of one combination to split it up by a new set of affinities into new combinations never before seen or reached, and which would have remained long unknown under the mere empirical researches of the older chemistry. The peculiar properties and reactions of salicylic acid as an antiferment producing a demand for it, the German chemists, Kolbe* and Lautemann, sought for an organic compound which from its elementary composition might be split or dissociated into the desired new compound, salicylic acid. This substance, whose molecule might be broken up, they found in Phenol, or the so-called Carbolic Acid, and it is a very curious circumstance—purely accidental, so far as this writer knows—that a substance of well and long established character as an antiferment should have offered to these chemists a molecular constitution so well adapted to be broken up into a still more powerful antiferment; for there is no relation whatever, either in composition, or chemical, or physical properties between carbolic acid and salicylic acid, except in their effects as antiferments, and the two may, so far as present knowledge extends, accomplish these effects by similar or by altogether different reactions. The agent which the German chemists selected to resolve the molecules of Phenol into other molecules, one of which should be salicylic acid, was carbonic acid carbonic anhydride, as it is called in the new chemistry. Thus, from the action of carbonic acid on carbolic acid, salicylic acid is produced; a process which is about as far from the original willow tree as a source of the acid as can well be imagined, and yet a process which is as much the result of human knowledge based upon human research as that by which LeVerrier and Adams discovered the planet Neptune. It appears that where Phenol or Cresol, and perhaps others of this class of phenols, are combined with an alkali metal, such as sodium or potassium, thus forming phenol-sodium (often called phenate of soda) for example, and well dried carbonic anhydride is passed through the dry powder of phenol-sodium heated to 100° to 250° C. = 212° to 482° F., the reaction occurs which produces salicylate of sodium and other compounds. The salicylate of sodium thus formed is dissolved in water and decomposed by hydrochloric acid, which, uniting with the sodium by superior affinity, sets free the salicylic acid in the form of crystals. These crystals are washed and re-crystallized from a hot solution, and when dried, form a crystalline powder of a light brown color, somewhat resembling in color the powder of pale cinchona bark. This is unbleached salicylic acid and is probably pure enough for almost all, if not for all, the purposes to

* Archiv. der Pharmacie (3) v. 5. p. 445, from Jour. fur Practische Chemie. Bd. 10, S. 89, and quoted in Ding. Polyt. Journ. Bd. 214, S. 132, and in Parm. Jour. and Trans. of London. Third Series, No. 231.

which the acid is at present applied to practical uses. The small proportion of coloring matter which it contains in this condition is held by it with great tenacity, and the further processes by which it may be obtained of various shades up to whiteness are so difficult, troublesome and expensive that they more than double the cost of production. This bleaching may be accomplished in various ways to a certain extent; but to get the acid quite white, Kolbe reommends that it be converted into an ether, and this ether be again decomposed. In the writer's practice no good plan of decolorizing has as yet been reached, and as the decolorizing has not yet been shown to be necessary or very useful, no great attention has yet been given to it. The acid imported from Germany at very high prices is occasionally quite white; but most of that sold at the more moderate prices of two or three dollars per ounce is of various degrees of whiteness, up to a very light cream color with a reddish tinge. These varying shades of color seem to show that bleaching processes, more or less effective, have been used with all the acid yet imported into this country; while, so far as known, none has been made here until the writer undertook it. Hence, the entirely natural or entirely unbleached acid has not, so far as known, been yet used to any considerable extent; and it is a mere reasoning process, based upon the quantity and qualities of the coloring matter in the well made unbleached acid, by which it is inferred that for most if not for all of its present uses, this is as good as the more or less bleached product. If the well made unbleached acid be found to subserve all the useful purposes to which the substance may be applicable, as is confidently expected by the writer; and if the substance should, even in moderate degree, realize the expectations of its importance in the arts and in medicine, as indicated by tho European authorities, the process of Kolbe will make it practically attainable in the necessary quantities at a far lower cost; whilst without some such process it would be of very limited use to mankind, whatever might be its powers. Whether bleached or unbleached, the acid is in minute broken acicular crystals which give it the appearance of a granular powder, soft and smooth under the pestle or knife, but somewhat rough or resinous when rubbed between the fingers. This powder is odorless and nearly tasteless. It has, however, a sweetish and astringent aftertaste with slight acridity in the fauces, but none in the mouth; and though tasteless, it leaves a disposition or inclination to expectorate, which continues for some time.

It is practically insoluble in cold water, but is very soluble in hot water; and the water of a hot solution retains when cold, in proportion to its coldness, from about one part in two hundred and fifty to one part in five hundred of the solution. The presence of various neutral salts in small proportion in the water render it far more soluble. Up to this time phosphate of sodium seems to have been chiefly used in Germany* to render it more soluble in water for medicinal purposes, and it is said that three parts of phosphate of sodium will render one part of the acid easily so uble in fifty parts of water. It is much more soluble in alcohol and ether than in water. It meltes at about 125° C. = 257° F†. In common with other similar acids it forms salts with the principal bases, but these seem thus far to be difficult to make, and their effects have not been investigated.

It is used for medical and surgical purposes either dry or in solution. When used dry it is sprinked on wounds, ulcers, or dressings in the form of very fine powder, in very small quantities, either simply powdered or mixed in various proportions with some diluent, such as starch. When used in simple solution, either for spraying surfaces or for washes or gargles, it is used in tepid solution of about one part to three hundred parts of water. Where stronger solutions are required for warhes, gargles, or to moisten dressings, one part of the acid and three parts of phosphate of sodium to fifty parts of water have been used. When applied to wounds, it appears immediately in the urine‡.

Its alleged advantages over all other antiseptics are: Frst, that it is far more powerful and effective in similar quantities; and, secondly, that it is, in all quantities necessary for complete effectiveness, entirely devoid of irritant action upon the living tissues. It is not caustic nor corrosive in any quantity, and never produces inflammation. In large quantities it may be irritant and painful, but yet rarely surpasses a stimulant effect, while it appears to be quite neutral in the very small quantities which are yet thoroughly effective. Thirdly, it is said to reach and prevent processes of decomposition which are beyond the reach of all other antiseptics or antiferments. These processes are of two kinds, namely: vital, or those in which living organisms have an important part, such as that produced by yeast and many of those which occur in putrefaction; and chemical, or those which occur independent of vitality, as the production of volatile oils in mustard and bitter almonds, the effect of diastase, etc. Now, while carbolic acid and other antiferments are azymotic, or completely arrest or prevent fermentations of the first kind, they are powerless with the chemical processes. Salicylic acid is said to be more effective with the vital ferments, and equally effective with the chemical. Fourthly, in quantities said to be thoroughly effective, it is entirely odorless and tasteless and harmless, whilst it has no poisoning effect in any reasonable quantity.

It prevents or arrests the souring of worts, washes and beers of the brewers, and prevents or arrests the putrefactive agencies which are so troublesome and destructive to the glue manufacturers; and these and similar trades have thus far seemed to be its

* Thiersch. Pharm. Centralhalle, Oct. 22, Nov. 5. † Watts' Chem Dictionary, Art: "Salicylic Acid." ‡ Thiersch, as above cited.

principal consumers. Separate portions of fresh milk set aside to become sour, one to which 0.04 per cent. of salicylic acid was added soured thiriy-six hours later than the other. Urine thus protected was on the third day still clear and free from ammoniacal odor.

Varying proportions of the acid added to accurately measured separate portions of sweet milk, and these carefully observed afterward until they sour—or, by the use of meat juice instead of milk, observed closely for signs of putrefaction—would offer good indications of the quantities required to arrest these varieties of fermentation.

Professor Thiersch, of Leipsic*, used it upon contused and incised wounds, and in operations, with excellent general results, destroying the fetid odor of cancerous surfaces and pyæmic ulcerations. To such uses this writer would add the suggestion that for washing out the cavities of the abdomen and chest after those operations which tend so strongly to septicæmia, solutions of salicylic acid would seem to offer very great advantages, should it prove to be as bland and unirritating as it is stated to be, and yet so effective.

Most of these statements are summed up from the periodical literature of continental Europe during the past six months, little having appered upon the subject in Great Britain, or in this country, and nothing having been done with it, so far as known, in either country.

In occasional paragraphs and allusions benzoic acid has been coupled with salicylic acid, as being only second to it in effectiveness as an antiferment, and with similar advantages.

These statements are collated and condensed here as being well worth attention in themselves and in their relations to the phenomena of septic poisoning as already known. But they have a new significance, or at least suggest to this writer a new train of thought, when viewed in connection with some researches now in progress and but just appearing in the periodical literature.

Experiments† were made upon animals by the injection of measured quantities of septic blood. The blood of a healthy animal was allowed to become putrid. Increasing doses of this were injected into healthy animals until the amount necessary to cause death was ascertained. This quantity proved to be large, the animals recovering from all the small doses. Blood from the animal whose death was caused by injections of putrid blood was injected in increasing doses into healthy animals until the fatal dose was reached, and this dose was found to be smaller than that which killed the first animal. The blood of the second dead animal was used on healthy subjects in the same way as that of the first, and proved fatal in still smaller quantity. The expertments were con-

---

* Pharm. Centralhalle, Nos. 44 and 45, 1874.

† Bergman, Panum, Davaine, Vulpian and Bouley—the latter researches in Bulletins de l'Acad. de Med. 1872, 1873, and Davaine, translated by Mary C. Putnam, M. D., in Archives of Scientific and Practical Medicine, by C. E. Brown-Sequard and E. C. Seguin, No 5, p. 649.

tinued upon the same plan until finally a point was reached when a very minute portion—a fraction of a drop, perhaps—from the last animal proved fatal to the next, with more violent toxic symptoms and a shorter course. The important indications of this series of experiments is, of course, the rapid accumulation of potency in septic poisoning. And the question put by this indication is not only as to how this potency accumulates, but also how to prevent and arrest it. Metro-peritonitis and common pyæmia would doubtless, unobstructed, accumulate potency in the same way without visible inoculation, and often do continue and accumulate even against the vigorous application of the best means of prevention yet known. No hypothesis can be constructed that will embrace the phenomena of septic poisoning, as they are now rapidly being investigated without including zymotic diseases and the cachexiæ, and none will account for the phenomena already observed without bringing it within the sphere of what is called, in some of its degrees or phases, fermentation· Hence, if the medical art is to keep pace with the progress of the physical sciences, physicians can not afford to pass by such articles as salicylic and benzoic acids when offered by chemistry, without investigating their effects upon disease, even though not one out of ten should repay the labor of investigation; for it is certainly in this direction of research that medicine must look with greatest hope of success to control those abnormal vital processes which so far may be modified but not stopped. For example: Suppose a primary syhilitic or cancerons sore, or a diphtheritic patch, or even a cachectic pulmonary infarction, while these are merely the localized phenomena of an external inoculation, or of an internal taint,—they must all be considered to partake of the nature of a fermentation, and by some such process invade the whole organism Then suppose an antiferment which, when applied to any surface. not covered by an impervious cuticle, very soon appears, unchanged, first in the blood and then in the secretions and excretions—the manifest logical antagonism of such substance to the diseased conditions become too important to be neglected, and the counsels of wisdom demand that its claims to such antagonism be disprovod before it be dismissed. The question as to what may become of the cancer-cell, or of the less tangible precedent cause of it, or of the bacteria, or the precedent conditions which increase their fertility, under the well directed influence of this class of agents, is, perhaps, the most important one in all medical science And just in proportion as accurate research develops agents of greater and greater power, will be the prospect of better success in treatment.

The phenols, especially the so-called carbolic and cresylic acids (Phenol and Cresol), were and must always remain to be most important additions to this class of agents, surpassing in power all that had been previously tried. And if now salicylic acid shall prove more potent than the phenols, the farther gain will be very great, and the researches upon it will again lead up toward future discoveries of still greater power.—*Read before the Medical Society of the State of New York.*

